# Proximity extension assay inflammatory profiling cannot distinguish the presence of residual C-peptide in patients with long-standing type 1 diabetes

**DOI:** 10.1007/s00592-025-02537-9

**Published:** 2025-06-05

**Authors:** Ebrahim Anvari, Per Lundkvist, Kailash Singh, Daniel Espes

**Affiliations:** 1 Department of Medical Sciences, Uppsala University, Uppsala, Sweden; 2 Department of Medical Cell Biology, Uppsala University, Box 571, Uppsala, 75123 Sweden; 3 Science for Life Laboratory, Uppsala University, Uppsala, Sweden

**Keywords:** Type 1 diabetes, C-peptide, Proximity extension assay, Biomarkers

## Abstract

**Objective:**

Many patients with long-standing type 1 diabetes (T1D) have remaining low levels of C-peptide, i.e. and indirect sign of remaining functional beta-cells. This study focused on identifying differences in immunological and inflammatory biomarkers in patients with longstanding T1D and remaining C-peptide.

**Research design and methods:**

Adult patients (*n* = 120) with long-standing T1D (≥ 10 years) and healthy controls (HC) (*n* = 50) were recruited at Uppsala University Hospital. Residual beta-cell function was determined with an ultrasensitive C-peptide ELISA under fasting conditions. T1D patients were divided into two groups (C-peptide positive vs. C-peptide negative). Using the OLINK Explore Inflammation proximity extension assay (PEA), 368 circulating immunological and inflammatory biomarkers were analyzed in plasma.

**Results:**

The three groups could not be distinguished by principal component analysis and when correcting for multiple testing we found no differences in circulating biomarkers. However, based on uncorrected p-values there were six biomarkers that were different when comparing all T1D patients with HC and eight markers that were different when comparing C-peptide positive vs. negative T1D patients.

**Conclusion:**

A wide inflammatory assay analysis cannot distinguish patients with longstanding T1D and remaining C-peptide from patients with a complete loss of C-peptide nor from HC.

**Supplementary Information:**

The online version contains supplementary material available at 10.1007/s00592-025-02537-9.

## Introduction

C-peptide is the most widely adopted biomarker for functional beta-cell mass in type 1 diabetes (T1D) and has been used for decades in clinical practice [1]. Traditionally, it has been used as an important piece of information in the diagnosis of T1D [2, 3] in combination with the presence of the autoantibodies anti-glutamic acid decarboxylase (GAD) [4–6] and anti-tyrosine phosphatase like insulinoma antigen 2 (IA-2) [7, 8]. C-peptide is also used as the gold standard surrogate marker of functional beta-cell mass in clinical research and has been used to predict both remission and future complications of T1D [9, 10]. More recently established ultrasensitive C-peptide assays have made it possible to detect very low levels of circulating C-peptide even in patients with longstanding T1D [11–13]. Recent data have provided support for that presence of residual C-peptide in longstanding T1D is associated with a lower risk of microvascular complications and severe hypoglycemia [14].

However, it is still unclear whether the remaining beta-cells in individuals with long-standing T1D survived the initial destruction or if they could represent a unique cohort of beta-cells undergoing regeneration and remodeling decades after diagnosis. Regardless of whether these cells have survived the initial insult or if they constitute a pool of beta-cells undergoing remodeling, they could potentially represent an optimal target for regenerative drugs aiming to restore beta-cell mass. In one of our previous studies, we found that individuals with long-standing T1D and remaining C-peptide also had higher circulating levels of the anti-inflammatory cytokine IL-35 and a higher proportion of IL-35 positive regulatory T cells [15]. Although these findings cannot answer the question of the origin of the remaining beta-cells, they still point in the direction towards a more tolerogenic immune profile in individuals with longstanding T1D and remaining C-peptide. However, another study in patients with longstanding T1D found that pro-inflammatory cytokines, such as TNF-α and IL-6, was associated with residual C-peptide, suggestive of an ongoing inflammatory process [16].

Considering the complexity and vast number of both pro- and anti-inflammatory cytokines and other mediators that are in play in parallel, we have in the current study applied a significantly wider inflammatory panel based on Proximity Extension Assay (PEA) technology in order to investigate if patients with long-standing T1D and remaining C-peptide display a distinctive inflammatory- and immunological profile.

## Research design and methods

### Ethical statement

The study was approved by the Regional Research Ethical Committee in Uppsala and was conducted in consistency with The Declaration of Helsinki. All participants gave their written informed consent prior to inclusion in the study.

### Study participants and blood sampling

Adult T1D patients (≥ 18 years of age) with ≥ 10 years of disease duration and age- and gender matched healthy volunteers were recruited at Uppsala University Hospital. Presence of diabetes complications were based on data from electronic medical records. Foot complications were, according to clinical routine in Sweden, based on clinical signs of neuropathy and/or peripheral vascular disease, pathologies of the skin and/or current or previous feet ulcers/amputations. Fasting blood samples were collected in the morning under standardized conditions. Routine lab parameters and auto-antibodies including GAD (limit value < 5 IE/mL) and IA-2 (limit value < 8 kE/L) were analyzed at the central clinical chemistry laboratory at Uppsala University Hospital according to clinical routine. EDTA-plasma was separated and immediately frozen in– 70 °C for later analysis of C-peptide and immunological/inflammatory biomarkers. Plasma C-peptide was analyzed blindly by Mercodia AB, Uppsala, Sweden, using an ultrasensitive ELISA (ultrasensitive ELISA, catalog no. 10-1141-01; Mercodia) with a lower detection limit of 1.17 pmol/L as previously described [15]. T1D patients were categorized as either C-peptide positive or negative based on the ultrasensitive C-peptide assay.

### Proximity extension assay (PEA)

Circulating immunological and inflammatory plasma biomarkers were determined by proximity extension assay (PEA) using the *Explore Inflammation Panel* (Olink AB, Uppsala, Sweden) and were analyzed at Olinks Laboratory in Uppsala according to the manufacturers protocol. A list of included biomarkers has been attached as a supplementary file.

### Correlation of residual C-peptide with circulating inflammatory biomarkers

In the group of C-peptide positive T1D patients we computed correlations of C-peptide levels with circulating normalized protein expression levels for all the analyzed biomarkers in order to investigate whether there is a correlation between the levels of C-peptide (i.e. a surrogate marker of residual beta-cell mass) and pro- or anti-inflammatory markers.

### Data preparation and statistical analysis

The software R (version 4.1.2) was used for data analysis, visualization and to compute the statistical analysis. A principal component analysis (PCA) was computed to examine the data for quality control (QC) warnings, outliers and to visualize how the samples from different groups separated from each other. An interquartile range (IQR) vs. median plot was constructed to inspect outliers after excluding QC warning-flagged samples. Three samples (one C-peptide negative and two C-peptide positive patients) were found to deviate and were therefore excluded. The remaining samples were inspected in another IQR vs. median plot which revealed another sample (healthy control) as an outlier. Following exclusion of that sample another IQR vs. median plot was constructed revealing no further outliers. After exclusion of samples flagged with QC warnings and outliers (*n* = 4 samples) there were in total *n* = 166 samples in the cohort out of which *n* = 49 were HC, *n* = 44 C-peptide positive and *n* = 73 C-peptide negative T1D patients which were included for statistical analysis.

For the final included samples, a PCA was computed and visualized as a plot. In the PCA each individual is represented by one point in the grid and the position is based on all measured protein values, i.e. all analyzed biomarkers are weighted into one point in the grid. The X- and Y-axis display the percentage of explained variance per principal component. An analysis of variance (ANOVA) was performed for comparison of the three groups (i.e. healthy, C-peptide positive and C-peptide negative) for each assay. Additionally, t-tests were performed for each assay for comparison between (1) healthy vs. all T1D patients and (2) C-peptide positive vs. C-peptide negative. P-values were corrected for multiple testing according to the Benjamini and Hochberg method. Corrected p-values < 0.05 were considered statistically significant and were given priority but also uncorrected p-values < 0.05 are presented. Correlation of circulating C-peptide levels in C-peptide positive patients and the normalized protein expression (NPX) levels was computed by using the spearman method.

## Results

### Descriptive data

In total, *n* = 120 patients with T1D and ≥ 10 years of disease duration and *n* = 50 healthy controls (HC) were recruited but since *n* = 4 samples were excluded from the PEA-analysis, as described above, these individuals were also excluded from the descriptive data since the PEA analysis was the main endpoint of the study. HC and T1D patients were comparable with regard to age, gender distribution and body mass index (BMI). Creatinine levels were comparable between the HC and T1D patients but as expected, HbA1c and fasting blood glucose was higher in the T1D group (Table 1). The age at onset was slightly higher in the group of T1D patients with remaining C-peptide when compared to those without (Table 1). Microvascular complications were common (especially retinopathy) in the T1D patients but was evenly distributed between the two subgroups (Table 2).

**Table 1:** Descriptive data of the healthy control (HC) group and patients with long-standing (> 10 years) type 1 diabetes (T1D) which were further subdivided into a C-peptide positive and negative group based on the detection of C-peptide with an ultrasensitive ELISA (detection limit 1.17 pmol/L)

Parameter	HC	T1D	*P*-value	C-peptide Negative	C-peptide Positive	*P*-value
Age (year)	38.5 ± 2.5	43.3 ± 1.4	0.12	40.7 ± 1.6	46 ± 2.4	0.06
Male gender, n (%)	24 (48.9%)	65 (55.5%)	0.39	38 (52%)	27 (61%)	0.1
BMI (kg/m2)	24.9 ± 0.7	25.4 ± 0.3	0.64	25.1 ± 0.4	25.7 ± 0.6	0.3
Debut age	–	16.3 ± 0.9	–	14.6 ± 1.1	18.2 ± 1.4	0.05*
Disease duration (year)	–	26.8 ± 1.2	–	26 ± 1.3	27.7 ± 2.2	0.5
B-Glucose (mmol/L)	5.5 ± 0.8	10.4 ± 0.4	< 0.001**	9.8 ± 0.5	10.9 ± 0.6	0.23
HbA1C (%, (mmol/mol))	5.2 (33.1 ± 0.5)	7.8 (61.8 ± 0.9)	< 0.001**	7.7 (61.2 ± 1.3)	7.8 (62.3 ± 1.3)	0.58
Creatinine (µmol/L)	75.3 ± 1.6	76.3 ± 1.9	0.77	76.0 ± 2.7	76.6 ± 2.6	0.88
eGFR (mL/min)	85.3 ± 1.2	88.8 ± 2.6	0.33	89.0 ± 3.3	88.7 ± 4.1	0.96
GAD positive (%)	0%	57.8%	< 0.001***	52%	63.6%	0.002***
IA-2 positive (%)	2%	32.3%	< 0.001***	26%	38.6%	< 0.001***
Positive for GAD and IA-2 (%)	0%	21%	< 0.001***	15%	27.2%	0.001***

Blood samples were collected in the morning after overnight fasting. Data are presented as mean ± SEM unless otherwise indicated. HbA1c is presented as mean values in NGSP % and within parenthesis as mean ± SEM in IFCC mmol/mol (as it was originally analyzed). P-values < 0.05 were considered statistically significant

*Denotes *p* < 0.05, ** <0.01 and *** <0.001

**Table 2:** Descriptive data of concomitant risk factors and presence of long-term complication in longstanding T1D patients that are either C-peptide positive or C-peptide negative

Parameter	C-peptide negative	C-peptide positive	*P*-value
Smoking, n (%)	9 (12%)	3 (6%)	0.31
Systolic blood pressure (mmHg)	121 ± 1.5	124 ± 2.1	0.2
Diastolic blood pressure (mmHg)	71.8 ± 1	71.3 ± 1.4	0.78
Hypertension, n (%)	21 (28%)	14 (30%)	0.8
Macroalbuminuria, n (%)	1 (1.3%)	0	0.42
Microalbuminuria, n (%)	9 (12%)	9 (19%)	0.26
Retinopathy, n (%)	57 (77%)	31 (67%)	0.24
Foot complication, n (%)	34 (46%)	25 (54%)	0.37

Microalbuminuria was defined as urine albumin concentrations of 20–300 mg/L and macroalbuminuria as > 300 mg/l. Foot complications were defined on the presence of clinical signs of neuropathy and/or peripheral vascular disease, pathologies of the skin and/or current or historical ulcers/amputations. Values are presented as number of individuals and within parenthesis as the percentage of the whole group. P-values < 0.05 were considered statistically significant. There was no significant difference in clinical microvascular complication between C-peptide positive and negative groups

### Principal component analysis

**Fig. 1:**
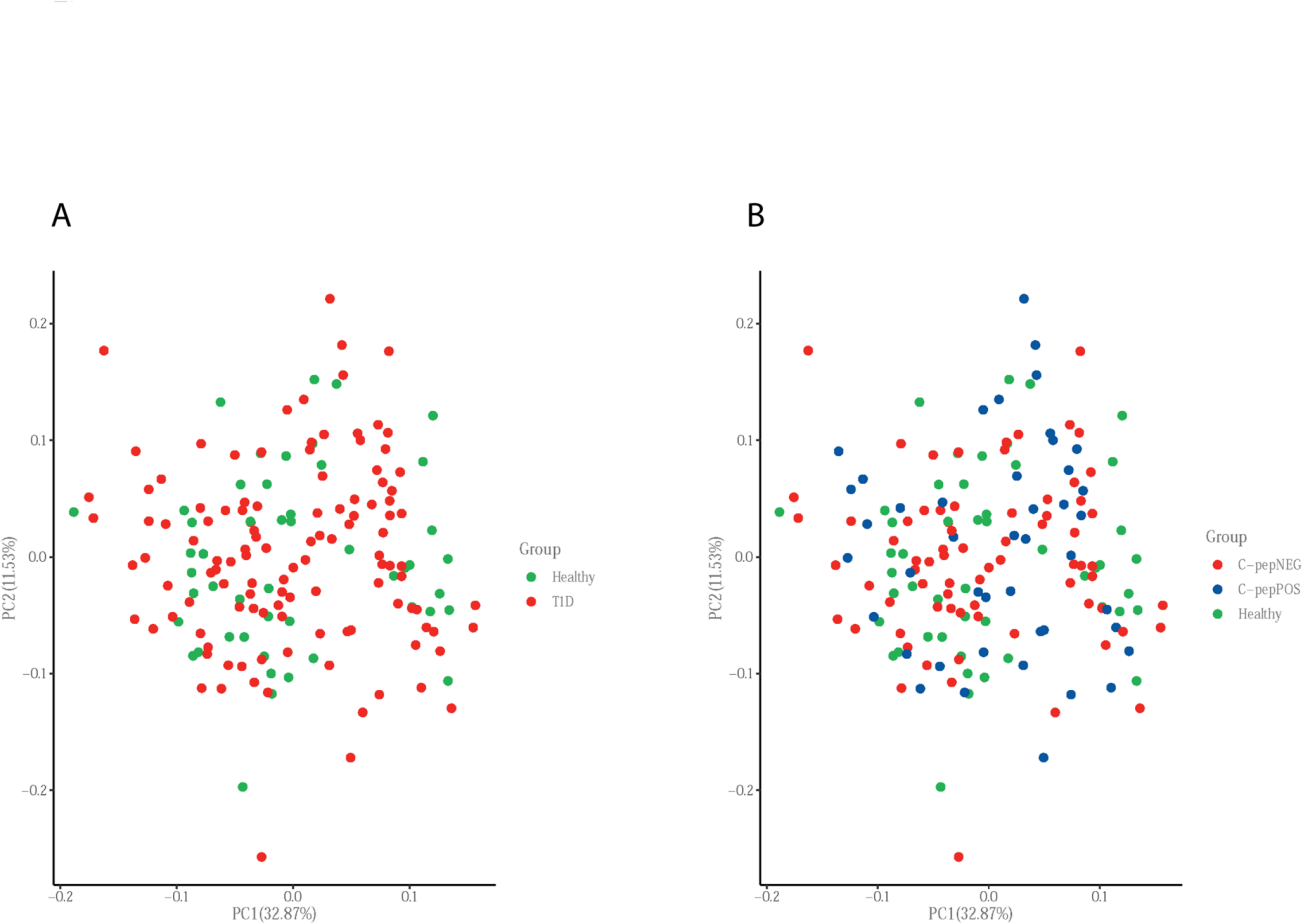
Principal component analysis (PCA) of the analyzed biomarkers. **A** PCA plot comparing healthy controls (green) and all patients with longstanding T1D (red), however no clear distinction between the two groups could be identified. **B** PCA plot comparing healthy controls (green), longstanding T1D patients with remaining C-peptide (blue) and those without remaining C-peptide (red). Again, no distinction between the groups was observed. Each point in the PCA-plots represents one and the position of the point is based on all measured protein values. The percentages displayed show the percentage of explained variance per principal component

Based on PCA plots, we could neither discriminate HC from T1D nor from C-peptide positive and C-peptide negative T1D patients (Fig. 1).

### Inflammatory biomarkers in T1D individuals and healthy controls

**Fig. 2:**
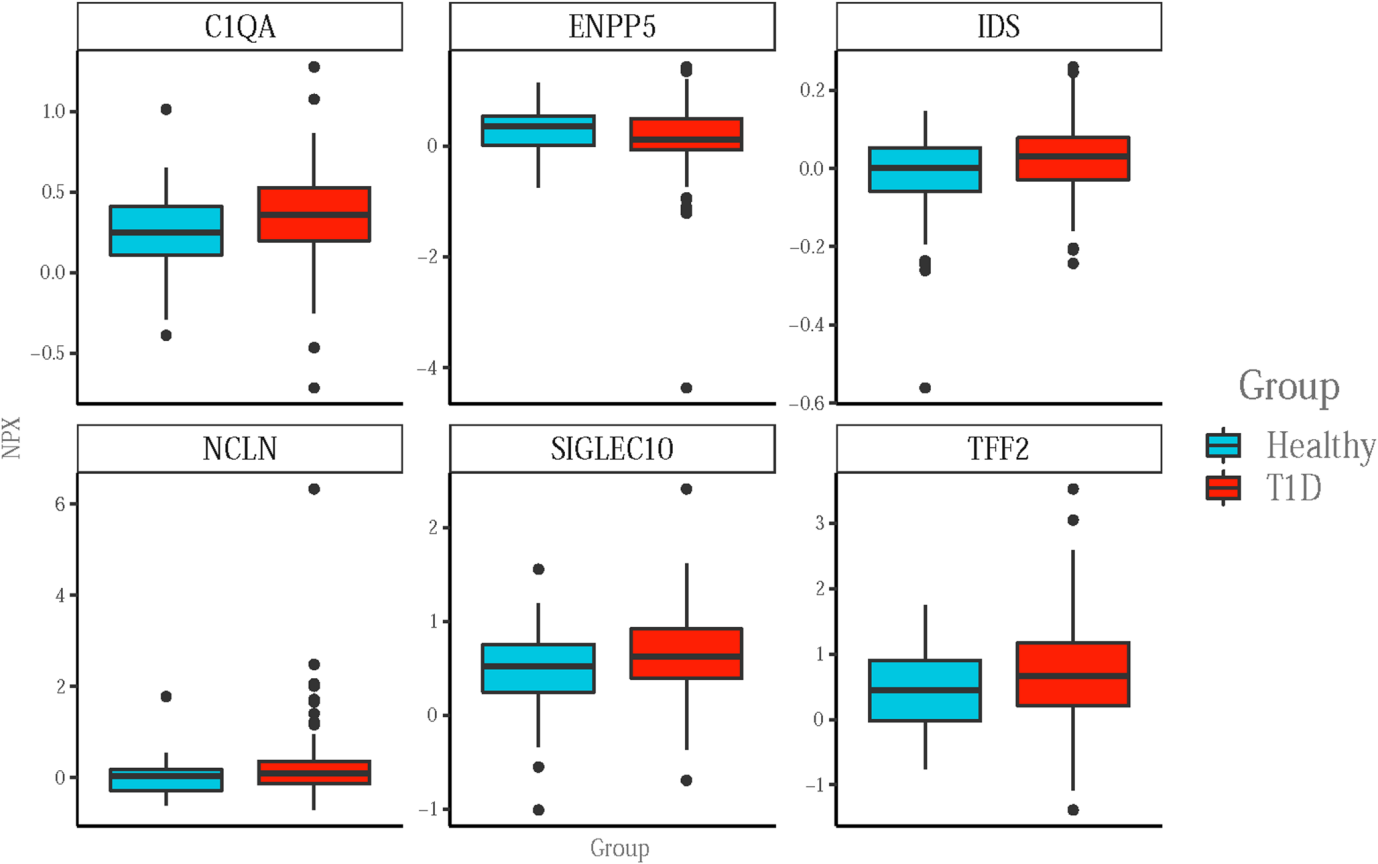
Comparison of individual biomarkers in healthy controls (HC) vs. longstanding type 1 diabetes (T1D). Boxplots of the top six assays in healthy controls vs. patients with longstanding T1D that were found to be significantly different based on uncorrected p-values. The boxplots show the distribution of data (NPX) for the grouping variable. The thick black horizontal line represents the median, the colored area - the interquartile range, the ends of the vertical middle line represent the minimum and the maximum values except for the black dots which are potential assay outliers

We found no difference in circulating biomarkers when comparing HC, C-peptide positive and C-peptide negative T1D patients, based on p-values corrected for multiple testing. Neither when comparing HC with all patients with T1D did we find any difference in the circulating inflammatory proteins based on p-values corrected for multiple testing. However, there were six biomarkers that differed between HC and T1D patients based on un-corrected p-values; Ectonucleotide Pyrophosphatase/Phosphodiesterase Family Member 5 (ENPP5) was lower in T1D than HC group while Complement C1q Subcomponent Subunit A (C1QA), Iduronate 2-Sulfatase (IDS), Niacilin (NCLN), Sialic Acid-binding Ig-like Lectin 10 (SIGLEC10) and Trefoil Factor 2 (TFF2) were higher in T1D than HC group (Fig. 2).

### Inflammatory biomarkers in C-peptide positive vs. negative T1D patients

**Fig. 3:**
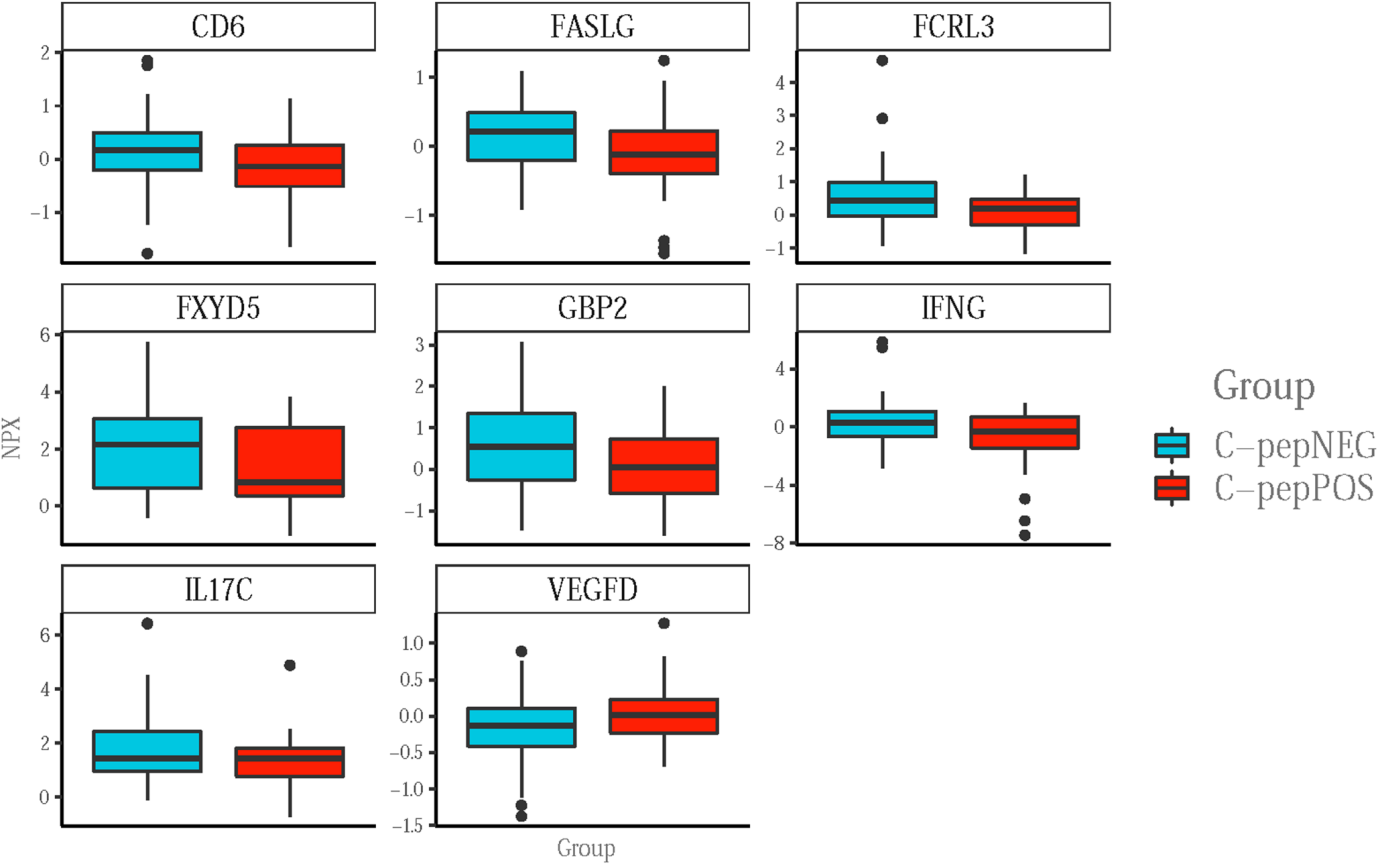
Comparison of individual biomarkers in patients with longstanding type 1 diabetes with- or without-remaining C-peptide. Boxplots of the top eight assays found to be significantly different based on uncorrected p-values in T1D patients with remaining C-peptide (red, C-pepPOS) compared to those without remaining C-peptide (blue, C-pepNEG). The boxplot shows the distribution of data (NPX) for the grouping variable. The thick black horizontal line represents the median, the colored area—the interquartile range. The ends of the vertical middle line represent the minimum and the maximum values except for the black dots which are potential assay outliers

None of the circulating inflammatory biomarkers differed between C-peptide positive and negative T1D patients when correcting for multiple testing. However, eight biomarkers differed based on uncorrected p-values, seven of these were lower in the C-peptide positive group; T cell Differentiation Antigen CD6 (CD6), Tumor Necrosis Factor Ligand Superfamily Member 6 (FASLG, a.k.a. FasL), Fc Receptor-like Protein 3 (FCRL3), FXYD Domain-containing Ion Transport Regulator 5 (FXYD5), Guanylate-binding Protein 2 (GBP2), Interferon Gamma (IFNG) and Interleukin 17 C (IL17C), while Vascular Endothelial Growth Factor D (VEGFD) was higher in the C-peptide positive group (Fig. 3).

### Correlation of residual C-peptide with circulating inflammatory biomarkers

In total, we found that there were 16 proteins that correlated with C-peptide levels (Table 3). Eight proteins had positive correlation and eight proteins had negative correlation with C-peptide levels. There was no overlap with the markers found to differ when comparing patients with remaining C-peptide to those without, or when comparing all T1D patients to HC.

**Table 3:** A correlation analysis was performed using the spearman method for the group of longstanding T1D patients that were C-peptide positive

Assay	Correlation	*P*-value
COL9A1	0.42	0.01
CCL26	– 0.41	0.01
NPPC	0.40	0.01
CCL4	– 0.37	0.01
SCG3	0.35	0.02
KLRB1	0.35	0.02
PTH1R	0.34	0.03
PKLR	– 0.33	0.03
OMD	0.32	0.03
FST	– 0.32	0.04
PPP1R9B	– 0.31	0.04
CCL11	– 0.31	0.04
NBN	– 0.32	0.04
CXCL1	– 0.31	0.04
PTX3	0.31	0.05
LY6D	0.30	0.05

This analysis aimed to show the relationship between two variables of normalized protein expression (NPX) and C-peptide levels as determined by an ultrasensitive C-peptide ELISA. The analysis found 16 markers that had a significant correlation with C-peptide levels

## Discussion

Inflammation is an important aspect of T1D pathology. In longstanding T1D, inflammation affects pancreatic beta-cells but it has also systematic implication related to complications. In this study, we aimed to identify differences in inflammatory biomarkers in patients with longstanding T1D and remaining C-peptide. For this purpose, we examined 368 inflammatory biomarkers and compared the presence between patients with longstanding T1D and healthy control, between the individuals with T1D with- and without remaining C-peptide and we examined the correlation of residual C-peptide with circulating inflammatory biomarkers.

Neither a PCA nor individual biomarker analyses could distinguish the groups from each other based on corrected p-value. Even based on uncorrected p-values there was only six biomarkers that differed when comparing all T1D patients with HC and eight that differed between C-peptide-positive and negative T1D patients.

Four of the six proteins that differed between the T1D and HC group based on uncorrected p-value have previously been found to play a role in microvascular complications. C1QA may play a role in the development of diabetic nephropathy [17], and NCLN can be upregulated in tubulointerstitial fibrosis, the hallmark of diabetic nephropathy [18], and IDS, a lysosomal enzyme involved in the degradation pathway proteoglycans, has been suggested to be involved in diabetes complications based on the important role of glycosaminoglycans (GAGs) degradation in both nephropathy and retinopathy [19, 20]. In our study, ENPP5 is the only protein that was found to be lower in T1D compared to HC. Downregulation of this protein has previously been identified in diabetic foot ulceration [21]. Hence, even though the inflammatory panel in the current study includes large number of biomarkers previously known to be related to inflammation in T1D, such as TNF-alpha, IL-6, IL-10, IL-32, none of these traditional markers were found to be altered in T1D when compared to HC. This may indicate that the main inflammatory pathology related to T1D per se is not as active anymore in long-standing T1D. Instead, the biomarkers that we found to be altered in this study seem to be more related to chronic microvascular complications in T1D. Albeit this was not the aim of the current study these markers could merit further studies aiming to identify early prognostic markers for identifying the risk of microvascular complications prior to clinical onset of tissue manifestations.

When comparing T1D patients with- and without remaining C-peptide we only found eight markers that differed based on uncorrected p-values. Interestingly, all of these markers, except for VEGF-D, were found to be slightly increased in the C-peptide negative group and have previously been investigated due to their potential role in beta-cell destruction, such as IFNG, FASLG and FCRL3 [22–24]. IFNG has an important role in T cell mediated autoimmunity and IFNG producing T cells (i.e. Th1 cells) are known mediators in autoimmune diseases, including T1D [25, 26]. It has also been demonstrated in separate studies, in both adults and children with T1D, that patients with lower endogenous insulin production (i.e. a reduced functional beta-cell mass) have higher concentrations of IFNG [23, 27]. This is in line with our finding that patients without remaining C-peptide have slightly higher levels of IFNG. FASLG, which has an essential role in CD8^+^ T cell mediated cytotoxicity, was also slightly higher in the C-peptide negative group. Many studies have found that FASLG plays a role in immune mediated pancreatic beta-cell destructions [22, 28–30] and recently, the role of FASLG in glucose induced apoptosis in beta-cells was described [31]. Therefore, the slightly elevated levels of FASLG observed in patients without remaining C-peptide may suggest that beta-cell loss could result from FASLG-induced beta-cell destruction. Additionally, a previous study from our group reported a reduced frequency of CD8^+^Foxp3^+^ Treg cells in patients without remaining C-peptide [15], which may be associated with higher FASLG levels, potentially contributing to the maintenance of the cytotoxic phenotype in CD8^+^ T cells.

FCRL3, a member of the immunoglobulin receptor superfamily, was also found to be slightly increased in the C-peptide negative patients. Although previous studies have not found an association with FCRL3 and adult onset T1D [32–34]. Pawlowicz et al. focused specifically on the distribution of selected FCLR3 and protein tyrosine phosphatase non-receptor type 22 (PTPN22) gene polymorphisms and their association with the progression of T1D in children with new-onset T1D [24]. They found a lower coincidence of the combination of certain genotypes of FCRL3 and PTPN22 in children with T1D compared to HC. Among T1D patients the genotype combination was found to be restricted to female patients and associated with a better-preserved endogenous insulin secretion during the two-year follow-up after diagnosis. In our study, PTPN22 was not included and FCRL3 was found to be slightly increased in C-peptide negative T1D patients.

We also found IL-17 C to be slightly increased in the C-peptide negative group. IL-17 has been presented as a pro-inflammatory cytokine in T1D [35, 36] but the mechanistic role of this cytokine is still unclear. However, the intracellular signaling of IL-17 C in immune cells involves activation of apoptosis pathways via B-cell Lymphoma 2 (Bcl-2), B-cell Lymphoma-extra Large (Bcl-XL) as well as the Nuclear Factor Kappa-light-chain-enhancer of Activated B-cells (NF-κB) and Mitogen-activated Protein Kinase (MAPK) [37]. Although highly speculative, this may play a role in the loss of residual beta-cell mass since we do find that the levels of IL-17 C are slightly higher in the T1D patients without remaining C-peptide. Consistent with this, we have also reported a higher frequency of IL-17a^+^ cells among Treg cells in patients without remaining C-peptide [15]. Together, our findings suggest that the proinflammatory IL-17 is elevated in patients lacking C-peptide, which may contribute to increased beta-cell apoptosis in these patients. In summary, of the markers that were found to be altered based on uncorrected p-values most were increased in C-peptide negative patients and these markers have previously been identified to be involved in beta-cell destruction. This could potentially reflect a remnant signature of the initial autoimmune attack despite the lack of functional endogenous beta-cells.

In the group of T1D patients with remaining C-peptide we computed correlations of C-peptide levels and the circulating inflammatory biomarkers. We found eight biomarkers that were negatively correlated with C-peptide levels and eight biomarkers that were positively correlated. Since these sixteen proteins neither overlapped with the biomarkers that were different between the two C-peptide subgroups nor between T1D and HC groups, it may suggest more generalized relations of these proteins with insulin rather than a specific role in the preservation or loss of beta-cells. For instance, Follistatin (FST), a glycoprotein found to be negatively correlated with C-peptide, has an important role in glucagon-insulin ratio and in healthy individuals, glucagon infusion has been observed to increases plasma FST significantly in combination with the expected suppression of insulin levels [38]. One of the biomarkers that had positive correlation with C-peptide in our study is Parathyroid Hormone 1 Receptor (PTH1R) which is primarily associated with calcium and bone metabolism. Interestingly, it has however also been shown that parathyroid hormone–related protein enhances human beta cell proliferation and function through the PTH1R [39]. Secretogranin III (SCG3) was also found to positively correlate with C-peptide in our study and SCG3 is known to play a role in the biogenesis of secretory granules in endocrine cells and to regulate insulin secretion [40]. We also found a number of chemokine biomarkers such as Chemokine (C-X-C motif) Ligand 1 (CXCL1), Chemokine (C-C motif) ligands 4 (CCL4), CCL11 and CCL26 to be negatively correlated with C-peptide levels. Chemokines and chemokine receptors and their role in the inflammatory processes involved in T1D have previously been studied [41]. CCL-4 (a Th2 related chemokine) has previously been found to negatively correlate with both proinsulin and, similar to our findings in longstanding T1D, with C-peptide levels one month after T1D debut [42]. Killer Cell Lectin Like Receptor B1 (KLRB1, a.k.a. CD161) has also previously been studied in T1D and, similar to our finding, found to be similar in both HC, longstanding T1D and even in patients with new onset T1D [43]. However, we did observe a positive correlation of KLRB1 and C-peptide which may indicate a more active autoimmunity in patients with remaining C-peptide, as CD8^+^CD161^+^ cells exhibit a heightened cytotoxic memory [44]. Although, interesting these correlations does not distinguish whether or not it represents an effect of insulin per se on immune cells or if it is an effect of remaining beta-cells. Also, as always with correlation there is a risk of identifying markers without a biological connection but there could also mirror the anabolic effects of insulin and its implication for other tissues rather than reflecting a role in the pathogenesis of T1D. Since it is known that insulin can stimulate the differentiation of cartilage and extracellular matrix deposition this could potentially explain the correlation with collagen alpha-1(IX) chain (COL9A1) and osteomodulin (OMD) [45]. Also, although there could be some biological relevance it is unlikely that the correlation with Nibrin (NBN), Neurabin-2 (PPP1R9B) and C-type natriuretic peptide (NPPC) reflects a prat of the pathogenesis with T1D associated with the preservation of C-peptide.

A strength of the current study is the use of PEA allowing for high sensitivity and large-scale studies of circulating biomarkers. In addition, we characterize both HC and patients with longstanding T1D with- and without remaining C-peptide. However, there are also a number of limitations to acknowledge. Firstly, the study is cross-sectional and blood samples were only collected at one time point under fasting conditions. Further, although intentional the use of a wide number of biomarkers does affect the read-out and introduces the risk of disregarding markers that could be of importance but failed to reach statistical significance due to the large number of included biomarkers. In order to mitigate this, we have also analyzed and reviewed the markers that were found to be altered based on uncorrected p-values. In addition, the current study is solely based on circulating levels of said biomarkers in plasma and were not further evaluated based on the secretion from isolated immune cells.

In conclusion, despite the extensive number of analyzed biomarkers, we cannot distinguish patients with longstanding T1D from HC, nor T1D patients with remaining C-peptide from those without. Even when considering uncorrected p-values, there are limited differences in circulating immune and inflammatory biomarkers in patients with longstanding T1D.

## Electronic supplementary material

Below is the link to the electronic supplementary material.

Supplementary Material 1

## Data Availability

Data will be made available upon reasonable request.

## References

[1] Jones AG, Hattersley AT (2013) The clinical utility of C-peptide measurement in the care of patients with diabetes. Diabet Med 30(7):803–817 23413806 10.1111/dme.12159 PMC3748788

[2] Landin-Olsson M, Nilsson KO, Lernmark A, Sundkvist G (1990) Islet cell antibodies and fasting C-peptide predict insulin requirement at diagnosis of diabetes mellitus. Diabetologia 33(9):561–568 2253834 10.1007/BF00404145

[3] Webb PG, Bonser AM (1981) Basal C-peptide in the discrimination of type I from type II diabetes. Diabetes Care 4(6):616–619 6751738 10.2337/diacare.4.6.616

[4] Solimena M, Folli F, Denis-Donini S, Comi GC, Pozza G, De Camilli P et al (1988) Autoantibodies to glutamic acid decarboxylase in a patient with stiff-man syndrome, epilepsy, and type I diabetes mellitus. N Engl J Med 318(16):1012–1020 3281011 10.1056/NEJM198804213181602

[5] Solimena M, Folli F, Aparisi R, Pozza G, De Camilli P (1990) Autoantibodies to GABA-ergic neurons and pancreatic beta cells in stiff-man syndrome. N Engl J Med 322(22):1555–1560 2135382 10.1056/NEJM199005313222202

[6] Solimena M, De Camilli P, Diabetes (1993) Spotlight on a neuronal enzyme. Nature 366(6450):15–17 8232529 10.1038/366015a0

[7] Bonifacio E, Lampasona V, Genovese S, Ferrari M, Bosi E (1995) Identification of protein tyrosine phosphatase-like IA2 (islet cell antigen 512) as the insulin-dependent diabetes-related 37/40K autoantigen and a target of islet-cell antibodies. J Immunol 155(11):5419–5426 7594559

[8] Solimena M, Dirkx R Jr., Hermel JM, Pleasic-Williams S, Shapiro JA, Caron L et al (1996) ICA 512, an autoantigen of type I diabetes, is an intrinsic membrane protein of neurosecretory granules. EMBO J 15(9):2102–2114 8641276 PMC450132

[9] Steffes MW, Sibley S, Jackson M, Thomas W (2003) Beta-cell function and the development of diabetes-related complications in the diabetes control and complications trial. Diabetes Care 26(3):832–836 12610045 10.2337/diacare.26.3.832

[10] Jeyam A, Colhoun H, McGurnaghan S, Blackbourn L, McDonald TJ, Palmer CNA et al (2021) Erratum. Clinical impact of residual C-peptide secretion in Type 1 diabetes on glycemia and microvascular complications. Diabetes Care 44:390–398. Diabetes Care. 2021;44(4):1072 10.2337/dc21-er04b 33731365

[11] Keenan HA, Sun JK, Levine J, Doria A, Aiello LP, Eisenbarth G et al (2010) Residual insulin production and pancreatic ss-cell turnover after 50 years of diabetes: Joslin medalist study. Diabetes 59(11):2846–2853 20699420 10.2337/db10-0676 PMC2963543

[12] Oram RA, Jones AG, Besser RE, Knight BA, Shields BM, Brown RJ et al (2014) The majority of patients with long-duration type 1 diabetes are insulin microsecretors and have functioning beta cells. Diabetologia 57(1):187–191 24121625 10.1007/s00125-013-3067-x PMC3855529

[13] Wang L, Lovejoy NF, Faustman DL (2012) Persistence of prolonged C-peptide production in type 1 diabetes as measured with an ultrasensitive C-peptide assay. Diabetes Care 35(3):465–470 22355018 10.2337/dc11-1236 PMC3322715

[14] Gubitosi-Klug RA, Braffett BH, Hitt S, Arends V, Uschner D, Jones K et al (2021) Residual beta cell function in long-term type 1 diabetes associates with reduced incidence of hypoglycemia. J Clin Invest 131(3). 10.1172/JCI143011 10.1172/JCI143011 PMC7843223 33529168

[15] Espes D, Singh K, Sandler S, Carlsson PO (2017) Increased Interleukin-35 levels in patients with type 1 diabetes with remaining C-Peptide. Diabetes Care 40(8):1090–1095 28620093 10.2337/dc16-2121

[16] Pham MN, Kolb H, Battelino T, Ludvigsson J, Pozzilli P, Zivehe F et al (2013) Fasting and meal-stimulated residual beta cell function is positively associated with serum concentrations of Proinflammatory cytokines and negatively associated with anti-inflammatory and regulatory cytokines in patients with longer term type 1 diabetes. Diabetologia 56(6):1356–1363 23494449 10.1007/s00125-013-2883-3

[17] Li Z, Feng J, Zhong J, Lu M, Gao X, Zhang Y (2022) Screening of the key genes and signalling pathways for diabetic nephropathy using bioinformatics analysis. Front Endocrinol (Lausanne) 13:864407 35923621 10.3389/fendo.2022.864407 PMC9340545

[18] Hsu YC, Chang PJ, Tung CW, Shih YH, Ni WC, Li YC et al (2020) De-Glycyrrhizinated licorice extract attenuates high Glucose-Stimulated renal tubular Epithelial-Mesenchymal transition via suppressing the Notch2 signaling pathway. Cells 9(1). 10.3390/cells9010125 10.3390/cells9010125 PMC7016866 31948095

[19] Hiebert LM (2021) Heparan sulfate proteoglycans in diabetes. Semin Thromb Hemost 47(3):261–273 33794551 10.1055/s-0041-1724118

[20] Kaur G, Song Y, Xia K, McCarthy K, Zhang F, Linhardt RJ et al (2022) Effect of high glucose on glycosaminoglycans in cultured retinal endothelial cells and rat retina. Glycobiology 32(8):720–734 35552402 10.1093/glycob/cwac029 PMC9280546

[21] Li X, Chen B, Xu Y, Zhou A, Wu B (2023) Identification of potential diagnostic biomarkers and immune infiltration features in diabetic foot ulcer by bioinformatics analysis and validation. Cell Mol Biol (Noisy-le-grand) 69(11):180–188 38015522 10.14715/cmb/2023.69.11.27

[22] Suarez-Pinzon WL, Power RF, Rabinovitch A (2000) Fas ligand-mediated mechanisms are involved in autoimmune destruction of islet beta cells in non-obese diabetic mice. Diabetologia 43(9):1149–1156 11043861 10.1007/s001250051506

[23] Kaas A, Pfleger C, Kharagjitsingh AV, Schloot NC, Hansen L, Buschard K et al (2012) Association between age, IL-10, IFNgamma, stimulated C-peptide and disease progression in children with newly diagnosed type 1 diabetes. Diabet Med 29(6):734–741 22150609 10.1111/j.1464-5491.2011.03544.x

[24] Pawlowicz M, Filipow R, Krzykowski G, Stanislawska-Sachadyn A, Morzuch L, Kulczycka J et al (2017) Coincidence of PTPN22 c.1858CC and FCRL3 -169CC genotypes as a biomarker of preserved residual beta-cell function in children with type 1 diabetes. Pediatr Diabetes 18(8):696–705 27615679 10.1111/pedi.12429

[25] Arif S, Tree TI, Astill TP, Tremble JM, Bishop AJ, Dayan CM et al (2004) Autoreactive T cell responses show Proinflammatory polarization in diabetes but a regulatory phenotype in health. J Clin Invest 113(3):451–463 14755342 10.1172/JCI19585 PMC324541

[26] Roep BO (2003) The role of T-cells in the pathogenesis of type 1 diabetes: from cause to cure. Diabetologia 46(3):305–321 12687328 10.1007/s00125-003-1089-5

[27] Alizadeh BZ, Hanifi-Moghaddam P, Eerligh P, van der Slik AR, Kolb H, Kharagjitsingh AV et al (2006) Association of interferon-gamma and Interleukin 10 genotypes and serum levels with partial clinical remission in type 1 diabetes. Clin Exp Immunol 145(3):480–484 16907917 10.1111/j.1365-2249.2006.03172.x PMC1809698

[28] Savinov AY, Tcherepanov A, Green EA, Flavell RA, Chervonsky AV (2003) Contribution of Fas to diabetes development. Proc Natl Acad Sci USA 100(2):628–632 12525697 10.1073/pnas.0237359100 PMC141047

[29] Mohamood AS, Guler ML, Xiao Z, Zheng D, Hess A, Wang Y et al (2007) Protection from autoimmune diabetes and T-cell lymphoproliferation induced by FasL mutation are differentially regulated and can be uncoupled Pharmacologically. Am J Pathol 171(1):97–106 17591957 10.2353/ajpath.2007.070148 PMC1941609

[30] Xiao Z, Mohamood AS, Uddin S, Gutfreund R, Nakata C, Marshall A et al (2011) Inhibition of Fas ligand in NOD mice unmasks a protective role for IL-10 against insulitis development. Am J Pathol 179(2):725–732 21718680 10.1016/j.ajpath.2011.04.016 PMC3157218

[31] Prasad MK, Mohandas S, Ramkumar KM (2023) Dysfunctions, molecular mechanisms, and therapeutic strategies of pancreatic beta-cells in diabetes. Apoptosis 28(7–8):958–976 37273039 10.1007/s10495-023-01854-0

[32] Duchatelet S, Caillat-Zucman S, Dubois-Laforgue D, Blanc H, Timsit J, Julier C (2008) FCRL3 -169CT functional polymorphism in type 1 diabetes and autoimmunity traits. Biomed Pharmacother 62(3):153–157 17961971 10.1016/j.biopha.2007.09.003

[33] Howson JM, Rosinger S, Smyth DJ, Boehm BO, Group A-ES, Todd JA (2011) Genetic analysis of adult-onset autoimmune diabetes. Diabetes 60(10):2645–2653 21873553 10.2337/db11-0364 PMC3178303

[34] Turunen JA, Wessman M, Kilpikari R, Parkkonen M, Forsblom C, Groop PH et al (2006) The functional variant– 169 C/T in the FCRL3 gene does not increase susceptibility to type 1 diabetes. Diabet Med 23(8):925–927 16911635 10.1111/j.1464-5491.2006.01848.x

[35] Abdel-Moneim A, Bakery HH, Allam G (2018) The potential pathogenic role of IL-17/Th17 cells in both type 1 and type 2 diabetes mellitus. Biomed Pharmacother 101:287–292 29499402 10.1016/j.biopha.2018.02.103

[36] Zheng Z, Zheng F (2019) A complex auxiliary: IL-17/Th17 signaling during type 1 diabetes progression. Mol Immunol 105:16–31 30472513 10.1016/j.molimm.2018.11.007

[37] Nies JF, Panzer U (2020) IL-17 C/IL-17RE: emergence of a unique Axis in T(H)17 biology. Front Immunol 11:341 32174926 10.3389/fimmu.2020.00341 PMC7054382

[38] Hansen JS, Rutti S, Arous C, Clemmesen JO, Secher NH, Drescher A et al (2016) Circulating follistatin is liver-derived and regulated by the Glucagon-to-Insulin ratio. J Clin Endocrinol Metab 101(2):550–560 26652766 10.1210/jc.2015-3668

[39] Guthalu Kondegowda N, Joshi-Gokhale S, Harb G, Williams K, Zhang XY, Takane KK et al (2010) Parathyroid hormone-related protein enhances human ss-cell proliferation and function with associated induction of Cyclin-dependent kinase 2 and Cyclin E expression. Diabetes 59(12):3131–3138 20876711 10.2337/db09-1796 PMC2992775

[40] Lin CC, Cheng KP, Hung HC, Li CH, Lin CH, Chang CJ et al (2019) Serum secretogranin III concentrations were increased in subjects with metabolic syndrome and independently associated with fasting plasma glucose levels. J Clin Med 8(9). 10.3390/jcm8091436 10.3390/jcm8091436 PMC6780385 31514320

[41] Pan X, Kaminga AC, Kinra S, Wen SW, Liu H, Tan X et al (2021) Chemokines in type 1 diabetes mellitus. Front Immunol 12:690082 35242125 10.3389/fimmu.2021.690082 PMC8886728

[42] Pfleger C, Kaas A, Hansen L, Alizadeh B, Hougaard P, Holl R et al (2008) Relation of Circulating concentrations of chemokine receptor CCR5 ligands to C-peptide, proinsulin and HbA1c and disease progression in type 1 diabetes. Clin Immunol 128(1):57–65 18434252 10.1016/j.clim.2008.03.458

[43] Kis J, Engelmann P, Farkas K, Richman G, Eck S, Lolley J et al (2007) Reduced CD4 + subset and Th1 bias of the human iNKT cells in type 1 diabetes mellitus. J Leukoc Biol 81(3):654–662 17151140 10.1189/jlb.1106654

[44] Konduri V, Oyewole-Said D, Vazquez-Perez J, Weldon SA, Halpert MM, Levitt JM et al (2020) CD8(+)CD161(+) T-Cells: cytotoxic memory cells with high therapeutic potential. Front Immunol 11:613204 33597948 10.3389/fimmu.2020.613204 PMC7882609

[45] Maor G, Silbermann M, von der Mark K, Heingard D, Laron Z (1993) Insulin enhances the growth of cartilage in organ and tissue cultures of mouse neonatal mandibular condyle. Calcif Tissue Int 52(4):291–299 8467410 10.1007/BF00296654

