# Supplementary material for: Proximity extension assay inflammatory profiling cannot distinguish the presence of residual C-peptide in patients with long-standing type 1 diabetes
Source: Acta Diabetol. 2025 Jun 5;62(11):1987–96. doi: 10.1007/s00592-025-02537-9 (PMC12640314; doi:10.1007/s00592-025-02537-9)
Supplement: Supplementary file 1 — Supplementary Material 1 [file 592_2025_2537_MOESM1_ESM.pdf]

Olink

Last updated: July 27, 2023

UniProt	Protein name	Gene name
P19878	Neutrophil cytosol factor 2	NCF2
P40933	Interleukin-15	IL15
P11274	Breakpoint cluster region protein	BCR
P52564	Dual specificity mitogen-activated protein kinase kinase 6	MAP2K6
Q9UN19	Dual adapter for phosphotyrosine and 3-phosphotyrosine and 3-phosphoinositide	DAPP1
P24394	Interleukin-4 receptor subunit alpha	IL4R
Q6ZUJ8	Phosphoinositide 3-kinase adapter protein 1	PIK3AP1
P01730	T-cell surface glycoprotein CD4	CD4
Q13241	Natural killer cells antigen CD94	KLRD1
P35613	Basigin	BSG
P50452	Serpin B8	SERPINB8
O43915	Vascular endothelial growth factor D	VEGFD
O00253	Agouti-related protein	AGRP
P10147	C-C motif chemokine 3	CCL3
Q92609	TBC1 domain family member 5	TBC1D5
Q9GZT9	Egl nine homolog 1	EGLN1
Q9Y266	Nuclear migration protein nudC	NUDC
Q14242	P-selectin glycoprotein ligand 1	SELPLG
Q12918	Killer cell lectin-like receptor subfamily B member 1	KLRB1
Q3KPI0	Carcinoembryonic antigen-related cell adhesion molecule 21	CEACAM21
Q9NRM6	Interleukin-17 receptor B	IL17RB
Q01344	Interleukin-5 receptor subunit alpha	IL5RA
P02745	Complement C1q subcomponent subunit A	C1QA
Q9HBG7	T-lymphocyte surface antigen Ly-9	LY9
O94992	Protein HEXIM1	HEXIM1
Q08174	Protocadherin-1	PCDH1

O60449	Lymphocyte antigen 75	LY75
O15455	Toll-like receptor 3	TLR3
P22304	Iduronate 2-sulfatase	IDS
P43234	Cathepsin O	CTSO
P14210	Hepatocyte growth factor	HGF
Q12866	Tyrosine-protein kinase Mer	MERTK
P51671	Eotaxin	CCL11
P42701	Interleukin-12 receptor subunit beta-1	IL12RB1
P09874	Poly [ADP-ribose] polymerase 1	PARP1
Q5R372	Rab GTPase-activating protein 1-like	RABGAP1L
Q13459	Unconventional myosin-IXb	MYO9B
O95760	Interleukin-33	IL33
P14784	Interleukin-2 receptor subunit beta	IL2RB
Q8NHJ6	Leukocyte immunoglobulin-like receptor subfamily B member 4	LILRB4
P01584	Interleukin-1 beta	IL1B
P60568	Interleukin-2	IL2
O76038	Secretagogin	SCGN
O95715	C-X-C motif chemokine 14	CXCL14
Q8N6P7	Interleukin-22 receptor subunit alpha-1	IL22RA1
P22301	Interleukin-10	IL10
Q9UPV0	Centrosomal protein of 164 kDa	CEP164
P28838	Cytosol aminopeptidase	LAP3
O60934	Nibrin	NBN
P57771	Regulator of G-protein signaling 8	RGS8
Q03426	Mevalonate kinase	MVK
O14904	Protein Wnt-9a	WNT9A
Q9Y478	5'-AMP-activated protein kinase subunit beta-1	PRKAB1
P20809	Interleukin-11	IL11
P05412	Transcription factor AP-1	JUN
O43707	Alpha-actinin-4	ACTN4
Q96PD4	Interleukin-17F	IL17F
P05112	Interleukin-4	IL4
P35225	Interleukin-13	IL13

Q96AX2	Ras-related protein Rab-37	RAB37
Q9NYY1	Interleukin-20	IL20
Q96P31	Fc receptor-like protein 3	FCRL3
Q9NP70	Ameloblastin	AMBN
Q13007	Interleukin-24	IL24
Q9HCU5	Prolactin regulatory element-binding protein	PREB
Q8WV07	Protein LTO1 homolog	LTO1
Q9Y2J8	Protein-arginine deiminase type-2	PADI2
Q9Y3P8	Signaling threshold-regulating transmembrane adapter 1	SIT1
Q8IU57	Interferon lambda receptor 1	IFNLR1
P30838	Aldehyde dehydrogenase, dimeric NADP-preferring	ALDH3A1
O14867	Transcription regulator protein BACH1	BACH1
P19801	Amiloride-sensitive amine oxidase [copper-containing]	AOC1
Q16552	Interleukin-17A	IL17A
Q7Z739	YTH domain-containing family protein 3	YTHDF3
O60575	Serine protease inhibitor Kazal-type 4	SPINK4
P26951	Interleukin-3 receptor subunit alpha	IL3RA
Q8TAD2	Interleukin-17D	IL17D
Q9P0M4	Interleukin-17C	IL17C
Q7Z6M3	Allergin-1	MILR1
Q8TCS8	Polyribonucleotide nucleotidyltransferase 1, mitochondrial	PNPT1
Q5T4W7	Artemin	ARTN
Q99748	Neurturin	NRTN
P48061	Stromal cell-derived factor 1	CXCL12
Q04759	Protein kinase C theta type	PRKCQ
Q12933	TNF receptor-associated factor 2	TRAF2
P42768	Wiskott-Aldrich syndrome protein	WAS
O95379	Tumor necrosis factor alpha-induced protein 8	TNFAIP8
Q13219	Pappalysin-1	PAPPA
Q13574	Diacylglycerol kinase zeta	DGKZ
P63241	Eukaryotic translation initiation factor 5A-1	EIF5A
O43736	Integral membrane protein 2A	ITM2A
O60542	Persephin	PSPN

P13693	Translationally-controlled tumor protein	TPT1
P09038	Fibroblast growth factor 2	FGF2
Q9Y5A7	NEDD8 ultimate buster 1	NUB1
Q6UXK5	Leucine-rich repeat neuronal protein 1	LRRN1
P01375	Tumor necrosis factor	TNF
Q13651	Interleukin-10 receptor subunit alpha	IL10RA
Q96RJ3	Tumor necrosis factor receptor superfamily member 13C	TNFRSF13C
P27540	Aryl hydrocarbon receptor nuclear translocator	ARNT
Q969V3	Nicalin	NCLN
Q9UHF4	Interleukin-20 receptor subunit alpha	IL20RA
Q06520	Bile salt sulfotransferase	SULT2A1
Q6UB28	Methionine aminopeptidase 1D, mitochondrial	METAP1D
Q0Z7S8	Fatty acid-binding protein 9	FABP9
O60880	SH2 domain-containing protein 1A	SH2D1A
Q12968	Nuclear factor of activated T-cells, cytoplasmic 3	NFATC3
P78362	SRSF protein kinase 2	SRPK2
P01903	HLA class II histocompatibility antigen, DR alpha chain	HLA-DRA
P78410	Butyrophilin subfamily 3 member A2	BTN3A2
O43521-2	Bcl-2-like protein 11, Isoform BimL	BCL2L11
P01583	Interleukin-1 alpha	IL1A
P01579	Interferon gamma	IFNG
Q05084	Islet cell autoantigen 1	ICA1
Q7L8A9	Tubulinyl-Tyr carboxypeptidase 1	VASH1
P05113	Interleukin-5	IL5
O43597	Protein sprouty homolog 2	SPRY2
Q13261	Interleukin-15 receptor subunit alpha	IL15RA
P12034	Fibroblast growth factor 5	FGF5
Q92844	TRAF family member-associated NF-kappa-B activator	TANK
O95644	Nuclear factor of activated T-cells, cytoplasmic 1	NFATC1
P09919	Granulocyte colony-stimulating factor	CSF3
Q9BXJ7	Protein amnionless	AMN
Q13291	Signaling lymphocytic activation molecule	SLAMF1
P51617	Interleukin-1 receptor-associated kinase 1	IRAK1

Q12778	Forkhead box protein O1	FOXO1
Q14435	Polypeptide N-acetylgalactosaminyltransferase 3	GALNT3
P30048	Thioredoxin-dependent peroxide reductase, mitochondrial	PRDX3
P32456	Guanylate-binding protein 2	GBP2
P01591	Immunoglobulin J chain	JCHAIN
P55957	BH3-interacting domain death agonist	BID
Q12765	Secernin-1	SCRN1
Q6ZMH5	Zinc transporter ZIP5	SLC39A5
Q8N8S7	Protein enabled homolog	ENAH
Q9Y6K9	NF-kappa-B essential modulator	IKBKG
P18564	Integrin beta-6	ITGB6
P58294	Prokineticin-1	PROK1
Q9HB29	Interleukin-1 receptor-like 2	IL1RL2
P05231	Interleukin-6	IL6
P12872	Promotilin	MLN
Q96DB9	FXYD domain-containing ion transport regulator 5	FXYD5
Q96LC7	Sialic acid-binding Ig-like lectin 10	SIGLEC10
O75475	PC4 and SFRS1-interacting protein	PSIP1
P19474	E3 ubiquitin-protein ligase TRIM21	TRIM21
B1AKI9	Isthmin-1	ISM1
P13232	Interleukin-7	IL7
P13747	HLA class I histocompatibility antigen, alpha chain E	HLA-E
Q9UNK0	Syntaxin-8	STX8
P33241	Lymphocyte-specific protein 1	LSP1
Q8WTT0	C-type lectin domain family 4 member C	CLEC4C
P13725	Oncostatin-M	OSM
Q8IVG5	Sterile alpha motif domain-containing protein 9-like	SAMD9L
Q8TD46	Cell surface glycoprotein CD200 receptor 1	CD200R1
Q9UHC6	Contactin-associated protein-like 2	CNTNAP2
P50995	Annexin A11	ANXA11
Q6DN72	Fc receptor-like protein 6	FCRL6
P23582	C-type natriuretic peptide	NPPC
Q8NDB2	B-cell scaffold protein with ankyrin repeats	BANK1

Q01151	CD83 antigen	CD83
P45984	Mitogen-activated protein kinase 9	MAPK9
Q9NRJ3	C-C motif chemokine 28	CCL28
Q9NZN5	Rho guanine nucleotide exchange factor 12	ARHGEF12
Q9HD26	Golgi-associated PDZ and coiled-coil motif-containing protein	GOPC
P28827	Receptor-type tyrosine-protein phosphatase mu	PTPRM
P29965	CD40 ligand	CD40LG
P16455	Methylated-DNA--protein-cysteine methyltransferase	MGMT
Q9BT73	Proteasome assembly chaperone 3	PSMG3
Q8N608	Inactive dipeptidyl peptidase 10	DPP10
P28845	Corticosteroid 11-beta-dehydrogenase isozyme 1	HSD11B1
Q9UNE0	Tumor necrosis factor receptor superfamily member EDAR	EDAR
P20849	Collagen alpha-1(IX) chain	COL9A1
Q9HCM2	Plexin-A4	PLXNA4
P01588	Erythropoietin	EPO
P23229	Integrin alpha-6	ITGA6
P80098	C-C motif chemokine 7	CCL7
O76036	Natural cytotoxicity triggering receptor 1	NCR1
P01374	Lymphotoxin-alpha	LTA
P42575	Caspase-2	CASP2
P24071	Immunoglobulin alpha Fc receptor	FCAR
Q9NWZ3	Interleukin-1 receptor-associated kinase 4	IRAK4
Q6UXB4	C-type lectin domain family 4 member G	CLEC4G
P37235	Hippocalcin-like protein 1	HPCAL1
Q9Y258	C-C motif chemokine 26	CCL26
Q9UKX5	Integrin alpha-11	ITGA11
Q9H0P0	Cytosolic 5'-nucleotidase 3A	NT5C3A
P08727	Keratin, type I cytoskeletal 19	KRT19
P20340	Ras-related protein Rab-6A	RAB6A
Q9UIB8	SLAM family member 5	CD84
P78310	Coxsackievirus and adenovirus receptor	CXADR
P32970	CD70 antigen	CD70
Q29983_Q29980	MHC class I polypeptide-related sequence A and MHC class I polypeptide-related sequen	MICA_MICB

O14788	Tumor necrosis factor ligand superfamily member 11	TNFSF11
Q9UDT6	CAP-Gly domain-containing linker protein 2	CLIP2
Q9C035	Tripartite motif-containing protein 5	TRIM5
P26022	Pentraxin-related protein PTX3	PTX3
Q07065	Cytoskeleton-associated protein 4	CKAP4
P80162	C-X-C motif chemokine 6	CXCL6
P20783	Neurotrophin-3	NTF3
Q14773	Intercellular adhesion molecule 4	ICAM4
Q16698	2,4-dienoyl-CoA reductase, mitochondrial	DECR1
P50591	Tumor necrosis factor ligand superfamily member 10	TNFSF10
Q8WXI8	C-type lectin domain family 4 member D	CLEC4D
O94856	Neurofascin	NFASC
P49771	Fms-related tyrosine kinase 3 ligand	FLT3LG
Q14005	Pro-interleukin-16	IL16
Q15517	Corneodesmosin	CDSN
O15169	Axin-1	AXIN1
Q9NQ25	SLAM family member 7	SLAMF7
Q9UMR7	C-type lectin domain family 4 member A	CLEC4A
O43561	Linker for activation of T-cells family member 1	LAT
P10145	Interleukin-8	CXCL8
Q96SB3	Neurabin-2	PPP1R9B
P41217	OX-2 membrane glycoprotein	CD200
P14317	Hematopoietic lineage cell-specific protein	HCLS1
Q9BZW8	Natural killer cell receptor 2B4	CD244
Q16719	Kynureninase	KYNU
O00273	DNA fragmentation factor subunit alpha	DFFA
Q13478	Interleukin-18 receptor 1	IL18R1
O75077	Disintegrin and metalloproteinase domain-containing protein 23	ADAM23
Q9UQV4	Lysosome-associated membrane glycoprotein 3	LAMP3
P24001	Interleukin-32	IL32
P36959	GMP reductase 1	GMPR
P30203	T-cell differentiation antigen CD6	CD6
P20273	B-cell receptor CD22	CD22

Q6UXB2	C-X-C motif chemokine 17	CXCL17
P68106	Peptidyl-prolyl cis-trans isomerase FKBP1B	FKBP1B
P12544	Granzyme A	GZMA
O95971	CD160 antigen	CD160
P43489	Tumor necrosis factor receptor superfamily member 4	TNFRSF4
P01137	Transforming growth factor beta-1 proprotein	TGFB1
Q15661	Tryptase alpha/beta-1	TPSAB1
Q04637	Eukaryotic translation initiation factor 4 gamma 1	EIF4G1
P48023	Tumor necrosis factor ligand superfamily member 6	FASLG
P40259	B-cell antigen receptor complex-associated protein beta chain	CD79B
Q03431	Parathyroid hormone/parathyroid hormone-related peptide receptor	PTH1R
Q9Y6Q6	Tumor necrosis factor receptor superfamily member 11A	TNFRSF11A
Q96LA5	Fc receptor-like protein 2	FCRL2
Q9BXN2	C-type lectin domain family 7 member A	CLEC7A
Q9H4D0	Calsyntenin-2	CLSTN2
P29460	Interleukin-12 subunit beta	IL12B
P42702	Leukemia inhibitory factor receptor	LIFR
Q99616	C-C motif chemokine 13	CCL13
P00813	Adenosine deaminase	ADA
P30044	Peroxiredoxin-5, mitochondrial	PRDX5
O60884	DnaJ homolog subfamily A member 2	DNAJA2
P15692	Vascular endothelial growth factor A	VEGFA
O43508	Tumor necrosis factor ligand superfamily member 12	TNFSF12
O15444	C-C motif chemokine 25	CCL25
P10144	Granzyme B	GZMB
P01135	Protransforming growth factor alpha	TGFA
P78556	C-C motif chemokine 20	CCL20
P03956	Interstitial collagenase	MMP1
P49763	Placenta growth factor	PGF
Q9BY76	Angiopoietin-related protein 4	ANGPTL4
O95750	Fibroblast growth factor 19	FGF19
O14836	Tumor necrosis factor receptor superfamily member 13B	TNFRSF13B
P46109	Crk-like protein	CRKL

Q03405	Urokinase plasminogen activator surface receptor	PLAUR
O43598	2'-deoxynucleoside 5'-phosphate N-hydrolase 1	DNPH1
Q9HCB6	Spondin-1	SPON1
Q9NQ30	Endothelial cell-specific molecule 1	ESM1
Q16651	Prostasin	PRSS8
O00468	Agrin	AGRN
P29350	Tyrosine-protein phosphatase non-receptor type 6	PTPN6
Q07325	C-X-C motif chemokine 9	CXCL9
Q9UII2	ATPase inhibitor, mitochondrial	ATP5IF1
Q9H008	Phospholysine phosphohistidine inorganic pyrophosphate phosphatase	LHPP
P19876	C-X-C motif chemokine 3	CXCL3
Q6UWV6	Ectonucleotide pyrophosphatase/phosphodiesterase family member 7	ENPP7
P09341	Growth-regulated alpha protein	CXCL1
Q9H3U7	SPARC-related modular calcium-binding protein 2	SMOC2
Q92583	C-C motif chemokine 17	CCL17
Q99538	Legumain	LGMN
O00182	Galectin-9	LGALS9
Q03403	Trefoil factor 2	TFF2
P53634	Dipeptidyl peptidase 1	CTSC
Q5ZPR3	CD276 antigen	CD276
P55773	C-C motif chemokine 23	CCL23
P25116	Proteinase-activated receptor 1	F2R
Q9NZC2	Triggering receptor expressed on myeloid cells 2	TREM2
P34896	Serine hydroxymethyltransferase, cytosolic	SHMT1
Q15389	Angiopoietin-1	ANGPT1
O00626	C-C motif chemokine 22	CCL22
O75888	Tumor necrosis factor ligand superfamily member 13	TNFSF13
P47712	Cytosolic phospholipase A2	PLA2G4A
Q15166	Serum paraoxonase/lactonase 3	PON3
Q14118	Dystroglycan	DAG1
Q9BZZ2	Sialoadhesin	SIGLEC1
Q8NFT8	Delta and Notch-like epidermal growth factor-related receptor	DNER
Q99685	Monoglyceride lipase	MGLL

O00585	C-C motif chemokine 21	CCL21
P19256	Lymphocyte function-associated antigen 3	CD58
P09326	CD48 antigen	CD48
Q5KU26	Collectin-12	COLEC12
Q6GTX8	Leukocyte-associated immunoglobulin-like receptor 1	LAIR1
P09603	Macrophage colony-stimulating factor 1	CSF1
P51888	Prolargin	PRELP
P16422	Epithelial cell adhesion molecule	EPCAM
P01133	Pro-epidermal growth factor	EGF
P02778	C-X-C motif chemokine 10	CXCL10
Q92484	Acid sphingomyelinase-like phosphodiesterase 3a	SMPDL3A
Q7KYR7	Butyrophilin subfamily 2 member A1	BTN2A1
O43291	Kunitz-type protease inhibitor 2	SPINT2
Q9Y6N7	Roundabout homolog 1	ROBO1
Q8WU39	Marginal zone B- and B1-cell-specific protein	MZB1
P35625	Metalloproteinase inhibitor 3	TIMP3
O43639	Cytoplasmic protein NCK2	NCK2
O76096	Cystatin-F	CST7
O00339	Matrilin-2	MATN2
O75462	Cytokine receptor-like factor 1	CRLF1
Q9UJU6	Drebrin-like protein	DBNL
Q15109	Advanced glycosylation end product-specific receptor	AGER
Q08334	Interleukin-10 receptor subunit beta	IL10RB
Q9HC38	Glyoxalase domain-containing protein 4	GLOD4
P21709	Ephrin type-A receptor 1	EPHA1
P27930	Interleukin-1 receptor type 2	IL1R2
Q9UJA9	Ectonucleotide pyrophosphatase/phosphodiesterase family member 5	ENPP5
Q9NZV1	Cysteine-rich motor neuron 1 protein	CRIM1
P15260	Interferon gamma receptor 1	IFNGR1
P21860	Receptor tyrosine-protein kinase erbB-3	ERBB3
P09238	Stromelysin-2	MMP10
Q9NR12	PDZ and LIM domain protein 7	PDLIM7
P11684	Uteroglobin	SCGB1A1

P18510	Interleukin-1 receptor antagonist protein	IL1RN
Q14210	Lymphocyte antigen 6D	LY6D
Q14116	Interleukin-18	IL18
P13236	C-C motif chemokine 4	CCL4
Q99435	Protein kinase C-binding protein NELL2	NELL2
P36941	Tumor necrosis factor receptor superfamily member 3	LTBR
P30613	Pyruvate kinase PKLR	PKLR
P55145	Mesencephalic astrocyte-derived neurotrophic factor	MANF
O00241	Signal-regulatory protein beta-1	SIRPB1
P29279	CCN family member 2	CCN2
P0DMV8	Heat shock 70 kDa protein 1A	HSPA1A
O00300	Tumor necrosis factor receptor superfamily member 11B	TNFRSF11B
Q8WXD2	Secretogranin-3	SCG3
O14773	Tripeptidyl-peptidase 1	TPP1
Q96KG7	Multiple epidermal growth factor-like domains protein 10	MEGF10
Q4KMG0	Cell adhesion molecule-related/down-regulated by oncogenes	CDON
O95866	Megakaryocyte and platelet inhibitory receptor G6b	MPIG6B
P56470	Galectin-4	LGALS4
O75563	Src kinase-associated phosphoprotein 2	SKAP2
P01127	Platelet-derived growth factor subunit B	PDGFB
Q96PL1	Secretoglobin family 3A member 2	SCGB3A2
O95633	Follistatin-related protein 3	FSTL3
Q9UKU9	Angiopoietin-related protein 2	ANGPTL2
Q9BYZ8	Regenerating islet-derived protein 4	REG4
P24387	Corticotropin-releasing factor-binding protein	CRHBP
Q99983	Osteomodulin	OMD
Q13232	Nucleoside diphosphate kinase 3	NME3
Q9Y3D6	Mitochondrial fission 1 protein	FIS1
P19883	Follistatin	FST
P15291	Beta-1,4-galactosyltransferase 1	B4GALT1
P12532	Creatine kinase U-type, mitochondrial	CKMT1A_CKMT1B
Q9UHX3	Adhesion G protein-coupled receptor E2	ADGRE2
Q9NQ76	Matrix extracellular phosphoglycoprotein	MEPE

Q6UXH1	Protein disulfide isomerase CRELD2	CRELD2
Q99895	Chymotrypsin-C	CTRC
P07148	Fatty acid-binding protein, liver	FABP1
Q16363	Laminin subunit alpha-4	LAMA4
Q92956	Tumor necrosis factor receptor superfamily member 14	TNFRSF14
P22466	Galanin peptides	GAL
Q8TEU8	WAP, Kazal, immunoglobulin, Kunitz and NTR domain-containing protein 2	WFIKKN2
Q8IYS5	Osteoclast-associated immunoglobulin-like receptor	OSCAR
O00175	C-C motif chemokine 24	CCL24
P25942	Tumor necrosis factor receptor superfamily member 5	CD40
P54317	Pancreatic lipase-related protein 2	PNLIPRP2
Q9BU40	Chordin-like protein 1	CHRDL1
